# Study on the Water Quality Characteristics of the Baoan Lake Basin in China under Different Land Use and Landscape Pattern Distributions

**DOI:** 10.3390/ijerph19106082

**Published:** 2022-05-17

**Authors:** Weixiang Ren, Xiaodong Wu, Xuguang Ge, Guiying Lin, Lian Feng, Wanqing Ma, Dan Xu

**Affiliations:** College of Urban and Environmental Sciences, Hubei Normal University, Huangshi 435002, China; daxian_ren@163.com (W.R.); gxg76@hbnu.edu.cn (X.G.); linguiying86@126.com (G.L.); afenglian@163.com (L.F.); mwq379@163.com (W.M.); xudan745@163.com (D.X.)

**Keywords:** Baoan Lake basin, land use, landscape pattern, water quality

## Abstract

Land use and landscape pattern highly affect water quality. Their relationship can assist in land-use management and improve land-use efficiency. In this study, a water quality survey of rivers and lakes was performed in 2020 to analyze the effects of land use and the landscape pattern on the water quality of the rivers and lakes in the Baoan Lake basin and is expected to provide a reference for land use planning. The results demonstrated that the effects of land use on water quality were generally higher during the dry season than during the wet season; however, the opposite was demonstrated for the landscape pattern index. Cropland and urban land were closely correlated with deteriorating water quality, with contributions to total nitrogen, total phosphorous, and ammonia nitrogen in the basin. The impact of the landscape pattern of the basin on water quality was controlled by the original land-use type. In addition, the landscape configuration formed different land-use types to produce different effects on water quality. The basin scale better explained the changes in water quality, especially for construction land, followed by the 250 m and 500 m scales in the buffer zone.

## 1. Introduction

The water environment of a basin, as a link between different natural environmental cycles, has an important effect on each component of the ecosystem [1,2]. People have been living near water since ancient times and settlement has been dependent on river and lake banks in most cases [3]. The water quality in rivers and lakes within a basin directly affects human activities, such as drinking water, irrigation, and industrial production [4,5].

Land use and landscape pattern determine the distribution patterns of the ecosystem and management styles of land use in the basins [6]. Land use and landscape pattern can dramatically affect biological activities, material-energy transfer and exchange, as well as the regional microclimate in basins [7,8]. Undoubtedly, land-use status is closely related to the water quality of the rivers and lakes in the basins [4]. The different distribution patterns of the same land type also exert different impacts on water quality in different basins [9]. In general, the relationship between land use, the landscape pattern, and the water quality of basins is quite complex and understanding the relationship between water quality and land use can assist policymakers in optimizing land management policies and improving land-use efficiency [10].

In terms of land use, the influence of cropland, forest land, grassland, the body of water, sandy land, and urban construction land on the water quality of rivers and lakes has been investigated [11,12]. Previous studies have included coastland and inland areas [12,13], low-altitude and high-altitude regions [14,15], as well as tropical and temperate regions [16,17]. In situ surveys and analytical studies have focused on certain basins. In addition, some analytical studies have been conducted for a target land type using methods to control the variables [11,18]. Most of these studies deduced that cropland and construction land are involved in the deterioration of water quality in rivers and lakes, whereas forest land and grassland help to alleviate pollution. However, other studies have revealed more complex patterns of related influences [19,20]. In terms of landscape pattern, researchers have deduced that both landscape configuration and landscape scale share different effects [19]. The landscape configurations that affect the water quality of rivers and lakes include the shape, size, number, density, and type of landscape patches; the length and curvature of the landscape corridors; and the proportion and distribution pattern of the landscape matrix [21,22]. For instance, He et al. used the space-for-time substitution method to analyze the temporal and spatial changes in the Lixia River landscape pattern, as well as their effects on water quality in the basin [23]. The landscape scale considers the target river segment and riverbank as the catchment, and a buffer range can be established along the riverbank to analyze which range scale better explains the change in water quality [24]. Studies on landscape configuration and scale remain uncertain, whereas different or even opposite results have been reported in different studies [25,26].

The Baoan Lake basin is located at the junction of Huangshi City and Ezhou City, Hubei Province, China. The main water bodies within the basin are Baoan Lake and Sanshan Lake. Agricultural production constitutes the main economic behavior in the Baoan Lake basin, where farmland and dikes are widely distributed. Agricultural activities aggravate pollution in the basin, resulting in deteriorating water quality and a low rate of high-quality standards in lakes [27]. In this study, the influence of land use and landscape pattern status on the water quality of rivers and lakes in different seasons and at different scales, is investigated in the Baoan Lake basin. The results will provide a reference for territorial spatial planning, management, and remediation of the Baoan Lake basin, as well as help to protect and restore the water environments in other basins.

## 2. Materials and Methods

### 2.1. Overview of the Study Area and Sample Locations

**Figure 1:**
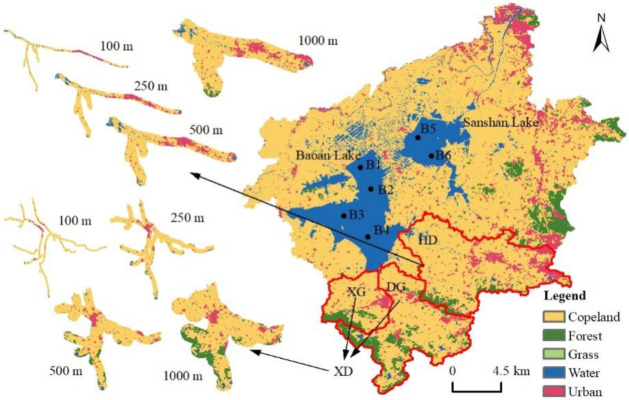
Overview of land use in the study area and a distribution map of the sampling locations. The main map on the right shows the scope of the Baoan Lake basin. The land in the basin is divided into five types: cropland, forest, grass, water, and urban. The water in the figure is Baoan Lake or Sanshan Lake. The scope marked by the red line on the lower side of the lake is the sub-basin selected for this study, in which Baoanxi Gang (XG) and Baoandong Gang (DG) are collectively referred to as XD. The small map on the left shows the 100–1000 m buffer zone range in the two sub-basins and its scale is consistent with the main map.

The Baoan Lake basin is located in the middle reaches of the Changjiang (Yangtze) River basin, which is in the subtropical monsoon climate zone, with an average annual precipitation of 1330 mm and average annual temperature of 16.8 °C. The perennial water surface areas of Baoan Lake and Sanshan Lake are 45.1 km^2^ and 20.2 km^2^, respectively. The inlet rivers of Baoan Lake mainly comprise the Huandiqiao Gang (HD River) in the southeast and the Baoanxi Gang (XG) and Baoandong Gang (DG) in the south. Part of the water of Sanshan Lake is fed by Baoan Lake during the flood season and the two lakes are linked by a river port. The lake water converges from the north side into the Changgang River, which flows into the Changjiang River [27]. In this study, six sample points were evenly set within Baoan Lake and Sanshan Lake (Figure 1). One sample point was set at each of the HD, XG, and DG inlets. Another reference point was set at the Xiushan Reservoir (HD0) at the source of the HD. The overall range of the XG and DG was referred to as XD for later analysis. According to the climate characteristics of the Baoan Lake basin, March-August was classified as the wet season and the remaining months were classified as the dry seasons.

### 2.2. Sample Collection and Measurement

Monthly filled surveys were conducted and samples were collected at the Baoan Lake basin in 2020. Mixed water samples were collected 50 cm below the water surface using a Plexiglas water picker and placed in acid-washed polyethylene bottles, which were immediately brought back to the laboratory for analysis. The indicators measured included pH, dissolved oxygen (DO), total nitrogen (TN), total phosphorous (TP), permanganate index (COD_Mn_), and ammonia nitrogen (NH_3_-N). Among them, pH and DO were measured using a multiparameter water quality monitor (YSI-EXO2), whereas the others were measured with reference to *Water and Wastewater Monitoring and Analysis Methods* [28].

### 2.3. Land-Use Data

The boundary of the Baoan Lake basin was extracted using DEM data at 30 m resolution. A Landsat 8 image for the summer of 2020 was downloaded from the United States Geological Survey website (https://earthexplorer.usgs.gov/; accessed on 15 January 2022) that covered the whole area of Baoan Lake and had clear image quality with less than 5% cloudiness. The land-use types were classified using the supervised classification method, and five land-use types were identified in the Baoan Lake basin, including cropland, forest land (forest), grassland (grass), water bodies (water), and urban construction land (urban).

### 2.4. Landscape Pattern Data

Based on the land-use classification data, the landscape pattern indices of the basin were calculated using the Fragstats 4.2 software. In this study, the indices included the patch density (PD), largest patch index (LPI), edge density (ED), landscape shape index (LSI), shape index (SHAPE), contiguity index (CONTIG), perimeter-area fractal dimension (PAFRAC), contagion (CONTAG), patch cohesion index (COHESION), landscape division index (DIVISION), Shannon’s diversity index (SHDI), and aggregation index (AI). The meaning and units of each index are shown in Table 1 [14,17].

### 2.5. Data Processing

The data were counted and plotted using Excel 2016 (Microsoft Corp., Redmond, WA, USA) and Origin 2020 software (OriginLab Corp., Northampton, MA, USA), including mean values, standard deviations, analysis of variance, and Pearson’s correlation coefficients. Two significant levels exist in the significance statement (*p* < 0.01 and *p* < 0.05), where *p* > 0.05 denotes insignificant.

**Figure 2:**
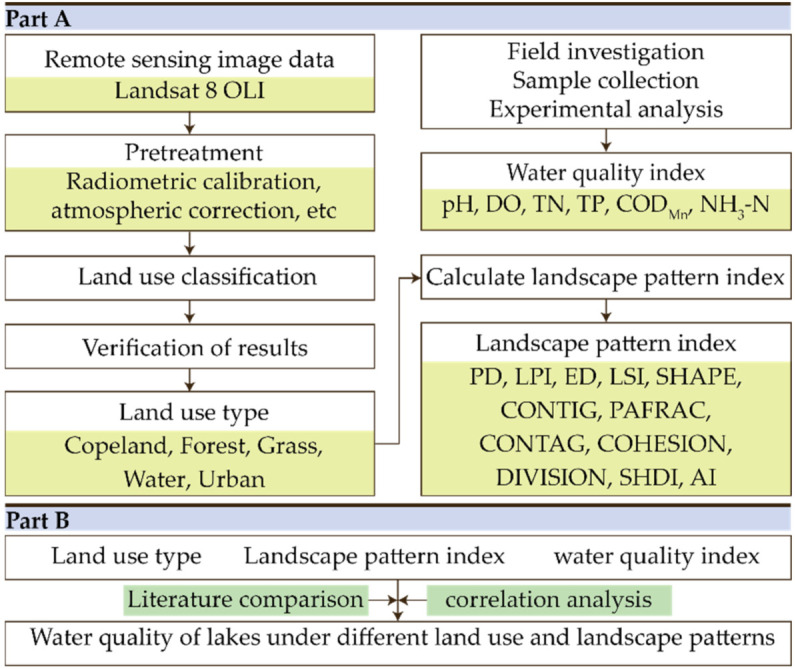
Technical framework. This research was divided into two parts. **Part A** included data acquisition and processing. The text with background color in the box is the data content required or developed during this step. **Part B** is the data analysis and discussion, and the text with background color is the method.

The overall technical framework of this study is shown in Figure 2.

## 3. Results

### 3.1. Status of Land Use and Landscape Pattern in the Baoan Lake Basin

**Figure 3:**
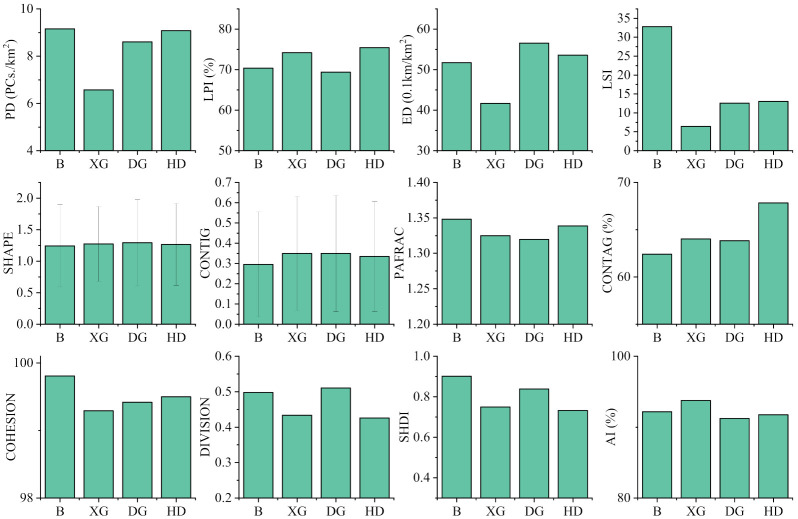
Distribution of the landscape pattern indices in the Baoan Lake basin and its sub-basins. In this figure, each index is patch density (PD), largest patch index (LPI), edge density (ED), landscape shape index (LSI), shape index (SHAPE), contiguity index (CONTIG), perimeter-area fractal dimension (PAFRAC), contagion (CONTAG), patch cohesion index (COHESION), landscape division index (DIVISION), Shannon’s diversity index (SHDI), and aggregation index (AI), respectively. B, XG, DG, and HD represent the Baoan Lake basin, Baoanxi Gang sub-basin, Baoandong Gang sub-basin, and Huandiqiao Gang sub-basin, respectively.

The distribution status of land use in the Baoan Lake basin and its sub-basins is shown in Table 2. The landscape pattern index status of each land type and basin is shown in Figure 3. The cropland was dominant within the basin, accounting for 71.18% of the whole basin, with a PD of 1.02/km^2^. Cropland was also uniformly distributed, maintaining a 70–75% land use ratio within the XG, DG, and HD sub-basins (Table 2). The land type with the least area was grassland, with only 0.01% of the land in the entire basin belonging to this type, which was primarily distributed in the built-up urban edges of the sub-basins of DG and HD (Figure 1). In addition, more than half the grassland was distributed within the sub-basin of DG (Figure 1). The range of the Baoan Lake basin was relatively small with the water body (mainly Baoan Lake and Sanshan Lake) accounting for 14.11% of the entire basin (Table 2). The forest land featured contiguous distributions in the eastern and southern parts of the basin (Figure 1), and the highest ratios of 20.15% and 14.15% were found in the sub-basin of DG and XG, respectively. However, the distribution of forest land in the sub-basin of XG was more concentrated (Table 2). The corresponding LPI value was 10.89% and the AI value was 92.87%, which were higher than those of all the other sub-basins and the entire Baoan Lake basin (Figure 3). The distribution of construction land was similar to that of forest land but with a wider range, accounting for 8.49% of the entire basin area. The distribution ratios of the construction land in the sub-basins of XG and DG were lower than that of forest land, but higher than that of the HD sub-basin, accounting for 14.49%, and the built-up urban area was also higher than that of the combined XG and DG sub-basins (Table 2). However, the most concentrated distribution of towns was within the XG sub-basin, with an LPI and AI of 5.44% and 81.12%, respectively. The distribution of towns in the HD sub-basin was more scattered, with a PD value of 4.82/km^2^ (Figure 3).

### 3.2. Water Quality Characteristics of the Lakes and Rivers Entering the Lake

**Figure 4:**
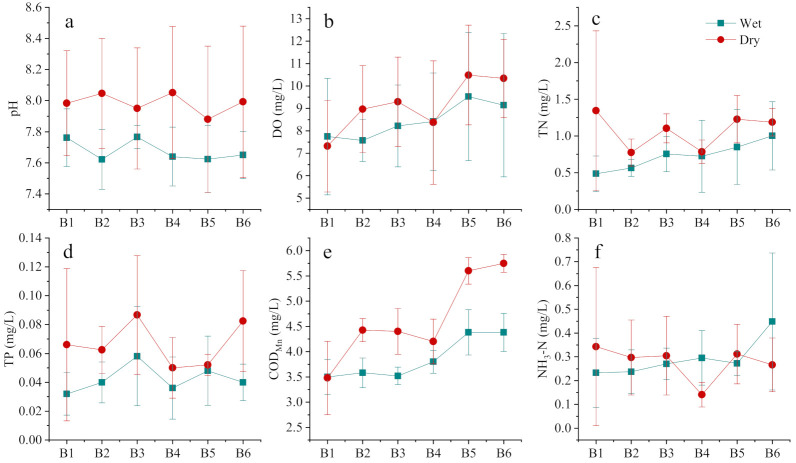
Distribution status of pH (**a**), DO (**b**), TN (**c**), TP (**d**), COD_Mn_ (**e**) and NH_3_-N (**f**) for Baoan Lake and Sanshan Lake during the dry and wet seasons. In this figure, B1–B4 are the sampling points in Baoan Lake, and B5–B6 are the sampling points in Sanshan Lake.

The distribution of most of the indicators was relatively smooth within Baoan Lake and Sanshan Lake. In terms of the dry–wet season change, the regularity of each indicator was gradually highlighted (Figure 4). More precisely, the pH values of the two lakes (7.98 ± 0.41 and 7.67 ± 0.17, respectively) were significantly higher during the dry season than during the wet season (*p* < 0.01). The DO was also higher during the dry season than the wet season within most of the areas of the lake, whereas the opposite trend occurred in Biandan Tang (Pond) (B1), which is located in the northern part of Baoan Lake. The TN, TP, and COD_Mn_ values were all higher during the dry season and lower during the wet season, with mean values of 1.07 ± 0.36 mg/L, 0.067 ± 0.029 mg/L, and 4.64 ± 0.38 mg/L during the dry season, respectively. TN reached the maximum of 3.38 mg/L during the dry season. The distribution of NH_3_-N during the dry and wet seasons was relatively uniform (*p* = 0.48).

**Figure 5:**
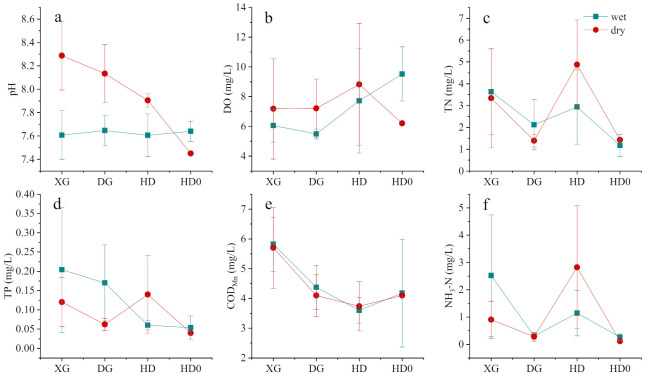
Differences in pH (**a**), DO (**b**), TN (**c**), TP (**d**), COD_Mn_ (**e**) and NH_3_-N (**f**) of the inlet rivers in Baoan Lake during the dry and wet seasons. In this figure, XG, DG, HD, and HD0 represent the Baoanxi Gang, Baoandong Gang, Huandiqiao Gang, and upstream points of Huandiqiao Gang, respectively.

A fluctuating pH was more evident in the XG and DG than in the HD sub-basin. This was attributed to the higher pH in the XG and DG sub-basins during the dry and wet seasons (Figure 5), both of which shared higher pH values during the dry season than during the wet season (*p* < 0.01). The DO level in the HD sub-basin was higher (15.1 mg/L) than in the XG and DG sub-basins (*p* < 0.05). The TN of the sub-basins had mean values of 2.75 ± 1.15 mg/L during the dry season and 2.47 ± 1.33 mg/L during the wet season (Figure 5). The TP in the sub-basins was concentrated throughout the year with a mean value of 0.075 ± 0.033 mg/L, whereas a high value of 0.500 mg/L occurred in the XG sub-basin. The difference in COD_Mn_ during the dry and wet seasons was not significant (*p* = 0.74), with the highest mean value of 5.74 ± 1.13 mg/L in the XG sub-basin and the lowest mean value of 3.67 ± 0.63 mg/L at the inlet of the HD sub-basin (Figure 5). The mean NH_3_-N level was 1.20 ± 0.80 mg/L throughout the year with a maximum of 6.32 mg/L in the HD sub-basin and a minimum of 0.09 mg/L in the DG sub-basin.

### 3.3. Relationship between Land Use, Landscape Pattern, and Water Quality in the Basin

**Figure 6:**
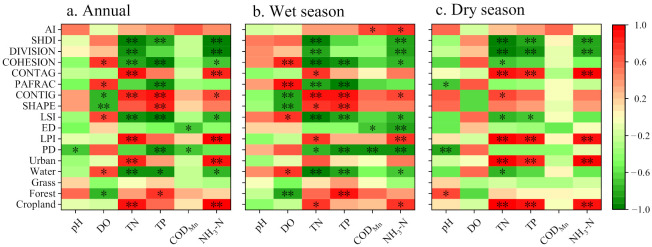
Relationship between land use, landscape pattern, and water quality in the Baoan Lake basin throughout the year (**a**), during the wet season (**b**), and the dry season (**c**). PD, LPI, ED, LSI, SHAPE, CONTIG, PAFRAC, CONTAG, COHESION, DIVISION, SHDI, and AI mean patch density, largest patch index, edge density, landscape shape index, shape index, contiguity index, perimeter-area fractal dimension, contagion, patch cohesion index, landscape division index, Shannon’s diversity index, and aggregation index, respectively. In this figure, a darker red indicates a higher degree of positive correlation, and a darker green indicates a higher degree of negative correlation. * and ** mean that the correlation was significant at *p* < 0.05 and *p* < 0.01, respectively.

The land types that most affected water quality at the basin scale were cropland, water, and construction land, and the latter two had greater impacts on nitrogen in rivers and lakes (Figure 6a, cropland: TN: r = 0.92, *p* < 0.01; NH_3_-H: r = 0.98, *p* < 0.01; construction land: TN: r = 0.84, *p* < 0.01; NH_3_-H: r = 0.89, *p* < 0.01). This trend was more prominent during the dry season (Figure 6c, cropland: TN: r = 0.97, *p* < 0.01; NH_3_-H: r = 0.92, *p* < 0.01; construction land: TN: r = 0.94, *p* < 0.01; NH_3_-H: r = 0.98, *p* < 0.01). In addition, the two land-use types also contributed more to TP in the basin during the dry season (Figure 6c, cropland: r = 0.91, *p* < 0.01; construction land: r = 0.85, *p* < 0.01). The contribution of cropland and construction land to the pollutants in the rivers and lakes during the wet season decreased compared to that during the dry season. However, the effects of forest land on TP (r = 0.90, *p* < 0.01) and DO (r = −0.86, *p* < 0.01) tended to be more prominent (Figure 6b).

The influences of different landscape pattern types on the input of different pollutants varied substantially (Figure 6). PD was negatively correlated with most of the indicators (Figure 6a, pH, COD_Mn_: *p* < 0.05; TP: *p* < 0.01), that is, the higher the patch density, the better the water quality. The LSI shared a similar effect with PD but with more of an effect on DO with a correlation coefficient of 0.70 (Figure 6a, *p* < 0.05). The SHAPE, CONTIG, and PAFRAC also had significant effects on DO (Figure 6a). The DIVISION index was negatively correlated with the two kinds of nitrogen in the basin (Figure 6a, *p* < 0.01). The effects of this indicator may be similar to that of PD. The ED was negatively correlated with COD_Mn_. However, this correlation was not clear during either the dry or wet season (Figure 6, *p* < 0.05).

The relationship between the landscape pattern indices and the water quality indicators during the dry season was closer than that during the wet season. PD, LSI, SHAPE, CONTIG, PAFRAC, and COHESION were correlated with more than three water quality indices (Figure 6b,c). In addition, AI was correlated with COD_Mn_ and NH_3_-N during the wet season, with correlation coefficients of 0.67 and 0.76 (Figure 6b, *p* < 0.05), respectively. The relationship between water quality and the landscape pattern was more obvious during the dry season. The three water quality indices (TN, TP, and NH_3_-N) were more closely correlated during the dry season (Figure 6c, all significance of *p* < 0.01; TN&TP: r = 0.91; TN&NH_3_-N: r = 0.94; TP& NH_3_-N: r = 0.84). Moreover, most of the landscape pattern indicators were strongly correlated with the three types of water quality indicators such as LPI, CONTAG, DIVISION, and SHDI. The PD was strongly correlated with most of the water quality indicators during the wet season. A similar case occurred for CONTIG.

**Figure 7:**
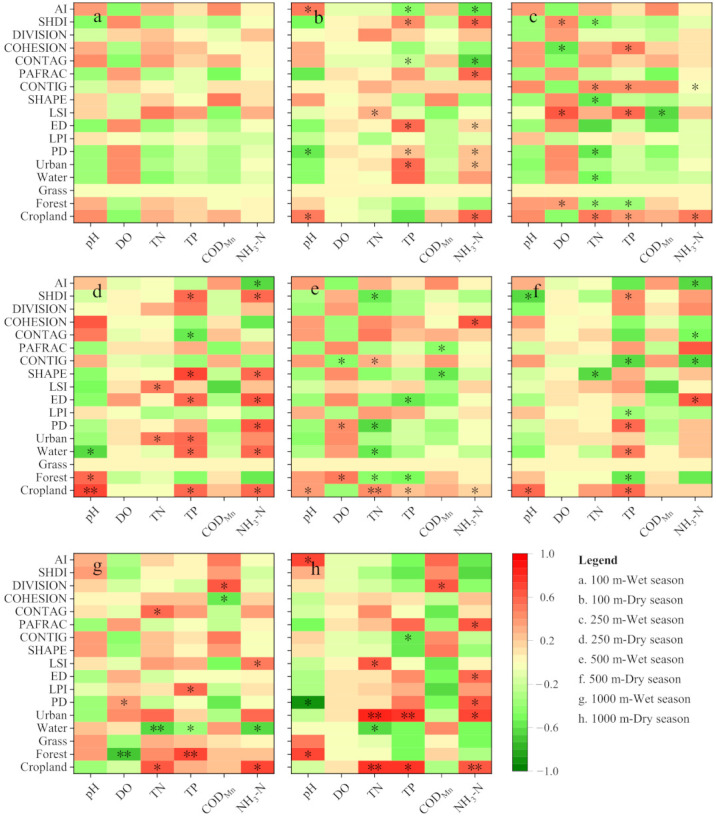
Relationship between land use, landscape pattern, and basin water quality during the dry and wet seasons, at different range scales. Scenarios include 100 m-wet season (**a**) and dry season (**b**), 250 m-wet season (**c**) and dry season (**d**), 500 m-wet season (**e**) and dry season (**f**), 1000 m-wet season (**g**) and dry season (**h**). PD, LPI, ED, LSI, SHAPE, CONTIG, PAFRAC, CONTAG, COHESION, DIVISION, SHDI, and AI mean patch density, largest patch index, edge density, landscape shape index, shape index, contiguity index, perimeter-area fractal dimension, contagion, patch cohesion index, landscape division index, Shannon’s diversity index, and aggregation index, respectively. In this figure, a darker red indicates a higher degree of positive correlation, and a darker green indicates a higher degree of negative correlation. * and ** mean that the correlation was significant at *p* < 0.05 and *p* < 0.01, respectively.

The impact of land use and landscape pattern on the water quality in the basin differed among the sub-basins at different range scales (Figure 7). During the wet season, the land use and landscape pattern status within the 100 m scale of the riverbank had no significant effects on the water quality of the river (Figure 7a, *p* > 0.05), whereas the 250 m and 500 m range scales had better effects. However, the overall effects were inferior to the entire basin scale (Figure 7c,e). At the 250 m and 500 m range scales, cropland and forest land were the main land-use types that highly affected water quality. No strong correlation was detected with construction land and in most cases, they were negatively correlated. Most of the landscape pattern indices were correlated with TN, such as PD, SHAPE, CONTIG, and SHDI (Figure 7c,e, *p* < 0.05). At the 1000 m range scale, the effects of land use on water quality were similar to the results generated at the entire basin scale, whereas the overall correlation was not as high as that of the entire basin scale (Figure 7g). In contrast, the relationship between the landscape pattern and water quality indicators at the 1000 m range scale was not strong. Most of the landscape pattern indicators were significantly correlated with a single water quality indicator. Similar results were observed for the 1000 m range scale during the dry season (Figure 7h).

During the dry season, most of the land use and landscape pattern indices were correlated with TP and NH_3_-N at the 100 m range scale, such as construction land, PD, ED, CONTAG, SHDI, and AI (Figure 7b). The relationship between the water quality status in the 500 m range scale during the dry season and the land-use status weakened compared to that during the wet season, whereas a stronger correlation was observed at the 250 m range scale (Figure 7d,f). At the two range scales, cropland and construction land remained the main land-use types affecting water quality in the basin, and most of the landscape pattern indices were significantly correlated with TP and NH_3_-N.

## 4. Discussion

### 4.1. Influence of the Land-Use Type on Water Quality in the Baoan Lake Basin

The two main land-use types affecting water quality in the Baoan Lake basin were cropland and construction land (Figure 6). More precisely, cropland constituted the most dominant land-use type in the Baoan Lake basin, with a distribution ratio exceeding 70% in the entire basin and each sub-basin. Cropland also played a matrix role in the overall landscape pattern distribution of the entire basin, with the LPI of the entire basin reaching 70.39% (Table 2 and Figure 3). The large area of cropland also contributed pollutants to the rivers and lakes. Previous studies have demonstrated that cropland is the surface source of pollution that is aggravating eutrophication in rivers and lakes [17,29]. The model developed by Alnahit et al. suggests that when the area of cropland within the basin exceeds 43%, the TN and TP in the rivers and lakes of the basin will increase significantly [30]. Nitrogen, phosphorous, organic matter, and water pH are significantly affected by cropland depending on the crop type, fertilizer type, and the local soil conditions [31,32,33]. Cropland mainly contributed nitrogen and phosphorous to the Baoan Lake basin that is transported into the lake and inlet rivers. The relationship between the two was stronger during the dry season than during the wet season (Figure 6), demonstrating that the surface-source sink flow of pollutants in the Baoan Lake basin cropland was not strongly correlated with the interflow surge from precipitation. On the one hand, although the amount of precipitation in the Baoan Lake basin was less during the dry season than that during the wet season, the precipitation remained regular, which ensures the full release of nitrogen and phosphorous in the cropland during each precipitation event. The interior cropland soil remained wet for long periods during the wet season, whereas nitrogen and phosphorous nutrients were diluted during the percolation process, thus indirectly weakening the relationship between them [34]. On the other hand, the growing season for crops in the Baoan Lake basin was mostly concentrated during the wet season. Large amounts of nutrients are absorbed by crops during the wet season, thus weakening the contribution of cropland to the pollutants in the rivers and lakes. The pollutants from crop harvesting and stalk decay were enriched during the dry season and were more likely to increase the contribution of cropland to the pollution in the rivers and lakes [35].

The contribution of urban construction land was similar to that of cropland, with TN, TP, and NH_3_-N as the main contributing pollutants, and the effects were stronger during the dry season than during the wet season (Figure 6). In contrast to cropland, the correlation between construction land and water quality differed significantly between the dry and wet seasons (Figure 6). The correlation between the cropland and water quality of the rivers and lakes was weaker during the wet season than during the dry season but a significant correlation was detected. Several water quality indicators were strongly correlated with the distribution of construction land during the dry season. However, the correlations were not significant during the wet season. Previous studies demonstrated that precipitation efficiently collects surface pollutants from impervious surfaces in urban areas and increases pollution in bodies of water after converging in the rivers and lakes [4,36,37]. However, in this study, precipitation was likely to show more complex effects on the water quality of the rivers and lakes in the urban areas. On the one hand, the precipitation in the Baoan Lake basin was more likely to dilute and weaken the contribution of construction land pollutants in the rivers and lakes, indicating that the towns in the Baoan Lake basin contribute more to the pollutants in the rivers and lakes through the pipe network. On the other hand, the impact of construction land on the water quality of rivers and lakes within the basin may be of larger spatial difference. Compared to cropland playing the “matrix” role within the basin, construction land was more scattered using a “patch” form in the basin. In this study, the HD and XD sub-basins were characterized by a high proportion of urban construction land, which accounted for 14.49% in the HD sub-basin (Table 2). They also had higher contributory levels of TN, TP, and NH_3_-N (Figure 6). The concentrations of TN, TP, and NH_3_-N in the HD sub-basin during the dry season were significantly higher than those during the wet season (Figure 4, *p* < 0.01), which was probably caused by the lower pollutant concentration during the wet season due to dilution by precipitation as previously analyzed. The differences in TN, TP, and NH_3_-N concentrations in the XD sub-basin between the dry and wet seasons were not significant. These indicators were slightly higher at some points during the wet season than the dry season. Therefore, the pollutant input by construction land in the XD sub-basin was more prone to being affected by precipitation.

Previous studies have demonstrated that forest land tends to be negatively correlated with the pollutant levels in rivers and lakes, acting as an interceptor and purifier of the pollutant input into rivers and lakes [9,38]. The results obtained by Rather et al. on the Dal Lake basin in India and by Zhang et al. on the Three Gorges Reservoir area, show that forests efficiently intercept nitrogen, phosphorous, and organic matter that sinks in lakes [15,39]. The relationship between forest land and the water quality of the rivers and lakes in the Baoan Lake basin was not strong, but forest land was positively correlated with TP during the wet season (Figure 6). The proportion of forest land in the Baoan Lake basin was not high, with the main distribution at the boundaries of the sub-basins (Figure 1). At the catchment scale of individual sub-basins, forest land was far away from the central catchment river. Furthermore, the overall terrain of the Baoan Lake basin was relatively flat and the input and interception of pollution for the forest land area remain in this area, which may be why forest land had less impact on water quality in the basin.

### 4.2. Influence of the Landscape Pattern on Water Quality in the Baoan Lake Basin

The size, density, shape, distribution pattern, and diversity of landscape patches can affect the water quality of a basin [10,40]. In this study, the PD, COHESION, DIVISION, and SHDI indices were negatively correlated with the water quality in the rivers and lakes. The LPI was positively correlated with water quality, which was similar to the results obtained by Wang et al. in Taihu Lake, China (Figure 6) [13]. However, several studies on the Danjiang River basin and the Three Gorges Reservoir area came to opposite conclusions. They reported that several indicators (such as PD and SHDI) indicate the fragmentation and diversity of the landscape in basins and that the use of fragmented and scattered land aggravates soil erosion and surface runoff, further increasing the pollutants in the rivers and lakes [22,39]. Such contradictory findings arise from the different land-use matrices in the different study areas. The land-use types of the landscape matrix within the Danjiang River and Three Georges Reservoir basins are forest land and grassland, whereas cropland and construction land are embedded as small, scattered patches. Therefore, the increase in patch fragmentation indicates an increase in non-point pollutant sources, which tend to contribute more pollutants [29,41]. However, in this study, the main land-use type in the Taihu Lake and Baoan Lake basins was cropland, whereas forest land was more embedded in patch form. Therefore, the increase in fragmented patches drives more pollutant effects, resulting in the negative correlation between the landscape pattern and water quality in the rivers and lakes. The larger LPI value caused by cropland contributed more pollutants to the rivers and lakes.

CONTIG reflects the connectivity between similar patch cell elements, whereas CONTAG reflects dispersed land use, with a higher value indicating a more concentrated land-use type. They were both positively correlated with the water quality in the rivers and lakes of the Baoan Lake basin (Figure 6), demonstrating that deteriorating water quality is closely correlated with more concentrated land-use types in the basin, that is, the landscape matrix type [42,43]. These findings are consistent with a previous analysis, which showed that cropland, as the most concentrated land type distributed in a basin, is closely related to deteriorating water quality.

In contrast to land use, the influence of the landscape pattern on water quality in the Baoan Lake basin was higher during the wet season than during the dry season, which is similar to the conclusions of Ruan et al. and Wu et al. [24,44]. The SHAPE and AI indices were positively correlated with water quality deterioration during the wet season (Figure 6), where SHAPE indicates the complexity of the patch shape and AI represents the agglomeration of land-use types. These combined results demonstrate that fragmented and complex land-use patterns tend to exacerbate deteriorating water quality, which provides a reference for regulating land use and management [45].

In summary, the landscape pattern status of a basin often affects the water quality of rivers and lakes by acting on the land-use types. The correlation between different landscape distribution types and water quality (either with contributory or antagonistic effects) was based on the original land type of the landscape pattern. The same landscape distribution pattern based on two different land-use types may have different effects on the water quality of the rivers and lakes [46].

### 4.3. Influence of Different Offshore Scales on Water Quality

The influence of land use and the landscape pattern status on water quality in basins at different range scales is often different, as reported previously [19]. For example, Mainali et al. demonstrated that land use and the landscape pattern better explain the 100 m buffer zone than the basin scale in the Han River basin in Korea [47]. Xu et al. performed multiple studies on the Yuan River basin at 100, 300, and 500 m range scales [48]. They deduced that the 300 m buffer zone scale best explained water quality. Several studies focusing on the Dongjiang River basin, the Sarapuí River basin, and the Oregon State River demonstrated that a basin scale better explains the changes in water quality than the riverbank scale [19,49,50]. In this study, we deduced that the basin scale would have the greatest effect on the water quality in the rivers and lakes, followed by the 250 m and 500 m range scales (Figure 7). The reason for the different results was likely related to the land management patterns in the different areas [22].

Different land-use types also differ in terms of their optimal range of influence on water quality in the basin [24]. In this study, the relationship between the water quality of the rivers and lakes and cropland and forest land was close at the 250 m and 500 m scales, whereas the relationship with urban construction land was weak, which is consistent with a previous study [51]. As reported previously, towns in the Baoan Lake basin are relatively concentrated in distribution, although the area accounts for a low proportion. Therefore, the contribution of towns to river and lake pollution is not uniformly distributed along the banks of the river and harbor. In contrast, cropland and forest land as matrices and patches embedded in the matrix produce a more uniform distribution effect in terms of their contributions to the water quality of the rivers and lakes in the offshore direction.

The effect of land use on the water quality in the rivers and lakes converged to that of the entire basin scale at a range scale of 1000 m (Figure 7). This occurred because the 1000 m buffer range covered most of the area in each sub-basin. However, the gap between the 1000 m buffer zone and the range of the sub-basin where it is located was often distributed with more forest land types. Therefore, the effects of forest land on water quality in the basin at a range scale of 1000 m may be underestimated. In addition, the relationship between the landscape pattern and water quality in the basin at a range scale of 1000 m was unclear because part of the landscape connection was directly cut when part of the buffer zone boundary converged at the basin boundary.

## 5. Conclusions

In this study, the effects of land use and landscape pattern status on water quality in the rivers and lakes of the Baoan Lake basin were analyzed in 2020 based on a field survey and monitoring. Cropland and construction land were closely correlated with deteriorating water quality in the rivers and lakes, and the correlations were stronger during the dry season than during the wet season. However, the influence of the landscape pattern status on water quality in the basin was stronger during the dry season than during the wet season. The mechanisms influencing river and lake water quality were different under different external conditions, so pollution prevention should not be carried out only for a certain land type or for a certain time.

Many previous studies evaluated the water quality based on the land-use type and landscape pattern of the original study area but did not discuss the effects of the various types of landscape patterns separately, so there were contradictions between the conclusions of different studies. The present study found that the effects of the landscape pattern in the basin were controlled by the original land-use type, whereas the landscape configuration patterns of different land-use types produced different effects.

Land use and the landscape pattern better explained the changes in water quality at the basin scale, particularly for the urban construction land type, followed by the 250 m and 500 m buffer scales. Therefore, land-use regulations on the banks of rivers and lakes should be considered when carrying out water environment treatment and restoration, and the restoration plan should be formulated from the river basin scale to improve the effects.

## Figures and Tables

**Table 1:** The landscape pattern index.

Name	Acronym	Describe	Unit
patch density	PD	Number of landscape patches per unit area.	PCs/km^2^
largest patch index	LPI	Proportion of the largest patch in the landscape area.	%
edge density	ED	The ratio of the total length of patch boundary to the landscape area.	0.1 km/km^2^
landscape shape index	LSI	The degree of deviation between the shape of a patch in the area and a circle or square of the same area	-
shape index	SHAPE	The mean value of the ratio between the perimeter of the patch and the square root of the area in the region.	-
contiguity index	CONTIG	Refers to the proximity between patches.	-
perimeter-area fractal dimension	PAFRAC	Calculation method of landscape patch regression degree.	-
contagion	CONTAG	Describes the degree of agglomeration of each patch type.	%
patch cohesion index	COHESION	Measures the physical connectivity of patch types.	-
landscape division index	DIVISION	The closer the value is to 1, the more serious the degree of landscape segmentation.	-
Shannon’s diversity index	SHDI	Measures the diversity of patch types in the landscape.	-
aggregation index	AI	Describes the degree of aggregation of spatial patterns.	%

**Table 2:** Land-use distribution in the Baoan Lake basin and its sub-basins.

	Cropland	Forest	Grass	Water	Urban
B	71.18%	6.19%	0.013%	14.11%	8.48%
XG	75.23%	14.14%	0.000%	0.50%	10.11%
DG	70.26%	20.15%	0.074%	1.33%	8.17%
HD	77.21%	6.38%	0.014%	1.89%	14.49%

Note: B, XG, DG, and HD represent the Baoan Lake basin, Baoanxi Gang sub-basin, Baoandong Gang sub-basin, and Huandiqiao Gang sub-basin, respectively.

## Data Availability

The datasets generated and/or analyzed during the current study are available from the corresponding author on request.
